# Coping strategies and resilient behavior among frontline healthcare workers: A scoping review

**DOI:** 10.1016/j.dialog.2025.100252

**Published:** 2025-10-23

**Authors:** Bilal Ahmad, Khalid Rehman, Shawana Bangash, Rida Zarkaish, Mahdia Babak, Sana Rehman, Samina Ihsan, Farhad Ali Khattak, Muhammad Irfan, Asif Rehman, Abdul Jalil Khan, Zohaib Khan

**Affiliations:** a Office of Research, Innovation and Commercialization, Khyber Medical University, Peshawar, Pakistan; b Institute of Public Health and Social Sciences, Khyber Medical University, Peshawar, Pakistan; c Khyber College of Dentistry (KCD), Peshawar, Pakistan; d Department of Psychiatry and Behavioral Sciences, Peshawar Medical College, Pakistan; e Riphah International University, Islamabad, Pakistan

**Keywords:** Coping strategies, Stress management, Frontline healthcare workers, Resilience, Mental health, Healthcare settings

## Abstract

This scoping review aimed to examine coping strategies and resilient behaviours for stress management among frontline healthcare workers as per the Sustainable Development Goal 3 (Ensure healthy lives and promote well-being for all). Five major databases (APA PsychInfo, Embase, MEDLINE, CINAHL, and Cochrane Library) were searched from their inception until December 2024, including peer-reviewed articles and the first three hundred results on Google Scholar. The review followed the JBI methodology for scoping reviews and PRISMA-ScR guidelines. The inclusion criteria were experimental and observational studies examining coping strategies and stress management interventions for frontline healthcare workers in any healthcare setting. Non-empirical studies, those without a clear methodology, and non-English-language studies were excluded. Data screening and selection were conducted using Rayyan software by two independent teams, with disagreements resolved through consensus with a third reviewer. Data extraction captured the study characteristics, population demographics, settings, and key findings. The cumulative sample size across all studies was 33,889 healthcare professionals, with significant gender disparity favouring female participants. This study spanned thirty countries across multiple continents, with the United States contributing the largest subset.

## 1 Introduction

Frontline Healthcare workers form the backbone of healthcare systems worldwide, providing essential services while often operating under significant pressure and challenging conditions [1]. The stress experienced by these professionals has long been recognised as a critical occupational health concern [2], but the recent global health emergencies, particularly COVID-19 pandemic, has brought unprecedented attention to the psychological challenges they face [3]. The impact of prolonged stress on healthcare workers manifests in various ways, including burnout, anxiety, depression, and reduced quality of patient care, making effective stress management crucial for both individual well-being and healthcare system functionality [1].

This review is in line with Sustainable Development Goal 3 (Ensure healthy lives and promote well-being for all), particularly Target 3.4 (promote mental health and well-being) and Target 3.8 (achieve universal health coverage) [4].

### 1.1 Evolution of healthcare worker stress

The nature of frontline healthcare workers' work inherently involves exposure to emotionally challenging situations, irregular schedules, high-stakes decision making, and complex interpersonal dynamics. These traditional stressors have been further exacerbated by contemporary healthcare challenges, including increased patient load, resource constraints, and rapidly evolving medical technologies [5]. Global health emergencies, such as the COVID-19 pandemic, further intensified these pressures, exposing frontline healthcare workers to additional physical risks, moral distress, and unprecedented uncertainty in their professional roles [6].

### 1.2 Impact on healthcare delivery

Research has shown that chronic workplace stress among healthcare workers can lead to severe consequences including decreased job satisfaction, increased medical errors, higher turnover rates, and compromised patient safety [7]. These outcomes not only affect individual healthcare workers but also have broader implications for healthcare system effectiveness and patient outcomes [8]. The relationship between healthcare worker well-being and quality of care creates an imperative to understand and address stress management effectively [9,10].

### 1.3 Current approaches to stress management

To address these challenges, various interventions and coping strategies have been developed and implemented across different healthcare settings. These range from individual-level approaches, such as mindfulness-based stress reduction [11] to organizational interventions focusing on workplace culture and support systems [12]. The effectiveness of these interventions appears to vary significantly based on contextual factors including resource availability, cultural considerations, and specific workplace demands [13].

### 1.4 Implementation challenges across resource setting

The implementation of stress management interventions is challenging in resource-limited settings due to various barriers [14], including lack of epidemiologic data, deficiencies in health systems organisation and resources, and institutional obstacles [14]. While high-income countries might have access to comprehensive support programs, healthcare workers in lower-middle-income and low-income countries often operate with minimal support systems despite facing similar or greater stressors [15]. This disparity highlights the need to understand how effective interventions can be adapted and implemented across resource settings.

### 1.5 Types of interventions

Frontline healthcare workers have faced significant stress during the pandemic, leading to various interventions aimed at supporting their mental health and well-being. Several types of interventions have been implemented and studied Psychological First Aid (PFA) has been utilized effectively, with clinicians trained in the Johns Hopkins RAPID model providing on-site support through daily well-being rounds [16]. This approach helped address acute crises and provided emotional support through active listening, mindfulness, validation, and cognitive interventions. Similarly, psychological first aid showed effectiveness across multiple studies with frontline workers [17].

Online interventions have also been developed, such as RESTORE (Recovering from Extreme Stressors Through Online Resources and *E*-health), which uses cognitive-behavioral techniques to address anxiety, depression, and PTSD symptoms [18]. This intervention demonstrated feasibility and significant improvements in mental health outcomes.

Other evidence-based interventions that have shown promise include eye movement desensitization and reprocessing (EMDR), trauma risk management, and various resilience-building programs [17]. Additionally, healthcare workers have employed self-care strategies such as mindfulness, gratitude practices, and seeking social support to build resilience [19].

Organizational-level interventions have also been implemented, including the establishment of Staff Support Centers, clinical treatment programs, team support sessions, and mental health and wellness programs [20]. However, research on organizational interventions in disaster settings is limited, indicating a need for further study in this area [21].

### 1.6 Study rationale and objectives

Despite the recognised importance of supporting healthcare workers' well-being and the existence of various intervention approaches, there has been no comprehensive mapping of stress management strategies across different healthcare contexts and economic settings. Understanding what works, for whom, and under what circumstances is crucial for developing effective context-appropriate interventions. This knowledge gap is particularly significant given the diverse nature of healthcare systems globally and the varying resources available for supporting the well-being of healthcare workers.

This scoping review aims to:

1. Comprehensively map the landscape of coping strategies and resilient behaviours among frontline healthcare workers.
2. Examine how these interventions are implemented across different economic settings.

## 2 Methods

### 2.1 Review framework

This scoping review followed the Preferred Reporting Items for Systematic Review and Meta-Analysis extension for Scoping Review (PRISMA-ScR) [22] guidelines. The protocol was registered in the Open Science Framework (OSF) Registry to ensure transparency and reproducibility.

### 2.2 Search strategy

#### 2.2.1 Information sources

We identified eligible papers by searching Medline, PsycINFO, and Embase via Ovid, CINAHL via EbscoHost, Cochrane Library, and Google Scholar (first 300 results) [23]. All searches were conducted from inception, with the final search strategy run on 27 November 2024.

#### 2.2.2 Search parameters

The key search terms are related to the:

• **Population** (i.e. frontline healthcare workers (nurses, healthcare workers, allied health professionals, and frontline workers))
• **Content** (i.e. stress management interventions and coping strategies), and context (i.e. any healthcare setting (hospitals, primary care, emergency services)).
• **Context** (i.e. Globally)

#### 2.2.3 Search strategy

The keywords for databases are (Frontline healthcare work*) OR (Healthcare work*) OR (allied health*) OR (nurse*) OR (medical doctor) AND (Stress manag*) OR (psycholog* resilient) OR (psycholog* resilien*) OR (Coping Stratag*) AND (Hospital) OR (healthcare facilit*) OR (healthcare setting) OR (Primary care) OR (Secondary care) OR (Tertiary care) and for search engine google scholar are “frontline healthcare workers” AND (“stress” OR “coping strategy”) AND (“primary care” OR “hospital”) (See supplementary material 1 for our full list of search terms).

#### 2.2.4 Selection criteria

We included English-language experimental studies (randomised controlled trials and quasi-experimental studies) and observational studies (cross-sectional, cohort, and case-control studies) that examined coping strategies and stress management interventions for frontline healthcare workers. We excluded non-empirical studies and those without a clear methodology.

### 2.3 Data management and selection process

#### 2.3.1 Data screening

We used Rayyan software for data screening and selection. Two independent teams conducted the screening process: Team 1 (RZ and SI) and Team 2 (MI and SR). Each team independently screened all titles and abstracts, and disagreements were resolved through consensus or consultation with a third independent reviewer (BA). For studies deemed potentially eligible or those with insufficient information in the abstract, the full texts were retrieved for further assessment. Full-text screening followed the same dual review process to determine the final study inclusion.

#### 2.3.2 Data extraction

Data extraction was independently conducted by two teams: Team 1 (RZ and SI) and Team 2 (MI and S). Any discrepancies in data extraction were resolved through consensus among the researchers, with a third author (BA) mediating when an agreement could not be reached. The data extraction framework begins with fundamental study characteristics, including author details, publication year, country of study, and research design methodology. This foundational information provided an essential context for understanding the research landscape. Additionally, we will document the journal of publication and any relevant funding sources or declare conflicts of interest to ensure transparency in the review process. Population demographics were carefully documented to understand the characteristics of healthcare workers. This includes detailed information about sample sizes, professional roles (such as nurses, physicians, or allied health professionals), work experience levels, and demographic distributions, including age and sex. These demographic data are crucial for identifying patterns in coping strategy effectiveness across different healthcare worker populations. The setting description captures the contextual elements of each study, including the type of healthcare facility, the specific departments or units involved, and whether the setting was urban or rural. This contextual information is vital for understanding how different environments influence the effectiveness of coping strategies. This information is particularly valuable for understanding how to effectively implement successful strategies in different healthcare contexts and lastly following the Peters et al. (2021) guidelines for narrative synthesis, two senior researchers (BA, ZK) independently analysed the extracted data [24].

### 2.4 Data analysis

#### 2.4.1 Analytical process

Our analysis followed a systematic narrative synthesis methodology following the guidance provided by Peters et al. (2021) [24], specifically designed to map coping strategies and resilient behaviours among frontline healthcare workers across different economic settings. The narrative synthesis methodology was selected as the most appropriate approach for addressing our research objectives, allowing for a comprehensive examination of how coping strategies vary according to economic context whilst preserving the contextual detail necessary for practical implementation guidance.

The synthesis process was systematically enhanced through integration with Claude 3.5 Sonnet [25], an advanced language model developed by Anthropic, to assist in managing and analysing the substantial volume of extracted data. This technological integration enhanced our ability to identify and organise recurring patterns related to coping strategies and resilient behavior among frontline healthcare workers across diverse economic settings.

#### 2.4.2 Data organisation and synthesis framework

Claude 3.5 sonnet version, a large language model developed by Anthropic [26]. Following comprehensive familiarisation with the dataset through extensive reading and re-reading of all included studies, data were systematically organised according to World Bank income classifications to examine how economic context influences coping strategy selection, adaptation, and effectiveness. Studies were categorized as high-income countries (HIC), upper-middle-income countries (UMIC), lower-middle-income countries (LMIC), and low-income countries (LIC) to facilitate systematic comparison across economic settings.

Within each economic classification, coping strategies were further organised according to their primary characteristics and implementation approaches. This included individual-level strategies such as mindfulness-based interventions, cognitive-behavioral approaches, and self-management techniques; social support mechanisms encompassing peer support, communication systems, and teamwork initiatives; organizational interventions including comprehensive programmes and institutional support systems; and technology-mediated interventions such as web-based programmes and mobile applications.

Claude 3.5 Sonnet assisted in this categorisation process by systematically identifying intervention types, outcome measures, and implementation characteristics across studies, whilst human researchers (BA, ZK) provided essential clinical interpretation and contextual understanding. This collaborative approach ensured that systematic pattern recognition was balanced with professional expertise and cultural sensitivity necessary for meaningful interpretation of findings.

#### 2.4.3 Narrative synthesis process

The narrative synthesis proceeded through systematic integration of findings within and across economic settings. Initial synthesis involved developing comprehensive descriptions of coping strategies employed within each income classification, identifying patterns in intervention selection, adaptation processes, and reported effectiveness. Particular attention was paid to how resource availability, healthcare system characteristics, and cultural factors influenced the implementation and adaptation of different coping strategies.

Cross-economic setting analysis examined similarities and differences in coping approaches, identifying both universal principles applicable across all settings and context-specific adaptations necessary for successful implementation in different economic environments. This analysis was supported by Claude 3.5 Sonnet's capacity for systematic comparison across the substantial dataset, whilst human researchers provided critical interpretation of clinical significance and practical implementation implications.

The synthesis specifically focused on mapping the landscape of coping strategies across economic settings, examining how interventions were adapted to local contexts, and identifying factors that contributed to successful implementation across diverse resource environments. This approach enabled comprehensive understanding of both the diversity and commonalities in healthcare worker coping strategies globally.

#### 2.4.4 Ensuring rigour

To ensure the rigour of our analysis, we employed several strategies:

1. **Researcher triangulation:** Two authors (BA and ZK) independently reviewed the codes and themes generated with the assistance of Claude 3.5 Sonnet, discussing and resolving any discrepancies.
2. **Comprehensive data inclusion:** We ensured that our analysis included a wide range of sources, including government reports, academic literature, and policy documents to provide a comprehensive view of social health protection schemes in Pakistan.
3. **Contextual consideration:** We paid particular attention to the unique context of Pakistan's healthcare system and the socio-economic factors that influence the implementation and effectiveness of social health protection schemes.

#### 2.4.5 Quality appraisal

1. As per the standard scoping review methodology, the included studies were not critically appraise the included studies [22]. The purpose of our scoping review was to identify and synthesise evidence regarding coping strategies, regardless of the methodological quality. This inclusive approach ensures that we capture the full range of available evidence and maintain alignment with the established scoping review guidelines. By focusing on evidence mapping rather than quality assessment, we can provide stakeholders with a comprehensive understanding of the current research landscape in stress management among healthcare workers.

AI-Human Analysis Comparison Table: Showing Classification Discrepancies of the studies by World Bank Income Levels.

**Table:** 

Finding Category	AI-Identified Studies				Human Identified Studies				Discrepancies Resolved	Final Studies			
	LIC	LMIC	UMIC	HIC	LIC	LMIC	UMIC	HIC		LIC	LMIC	UMIC	HIC
Social Support, communication and self-management	0	12	8	13	0	−3	−2	+5	AI incorrectly grouped LMIC interventions with UMIC settings; humans distinguished peer support networks specific to resource levels and resolved them through manual verification of economic classifications.	0	9	6	18
Cognitive behavioral intervention and mindfulness-based approaches	0	10	4	12	0	−2	−2	+2	AI combined formal CBT programmes across LMIC and UMIC; humans separated structured interventions from adapted mindfulness practices; resolved by distinguishing programme intensity and resource requirements.	0	8	2	14
Organizational and institutional approaches	0	3	0	2	0	−1	0	0	AI initially classified institutional programmes from LMIC settings; humans validated that these were organizational-level interventions; resolved through consensus on institutional capacity definitions.	0	2	0	2
Comprehensive multi-level program, creative and expressive approaches	0	2	1	6	0	−2	−1	+2	AI scattered multi-level programmes across income categories; humans identified these as primarily HIC resource-intensive interventions; resolved by resource availability analysis.	0	0	0	8
Technology-based intervention	1	0	0	5	0	+1	0	−1	AI missed mobile-based interventions in LMIC settings, focusing only on sophisticated web platforms; humans identified basic digital tools and smartphone apps, and resolved them through an expanded technology definition	1	1	0	4
Total	1	27	13	38	0	−7	−5	+8		1	20	8	46

### 2.5 Rigour enhancement through AI-human collaboration

#### 2.5.1 Artificial intelligence contributions to analytical efficiency

The integration of Claude 3.5 Sonnet demonstrated significant advantages in processing large-scale datasets efficiently whilst maintaining systematic consistency. The artificial intelligence system excelled at identifying coping strategies across different World Bank income classifications within our 75 included studies, substantially reducing the time required for initial pattern recognition compared to traditional manual coding approaches. Claude's capacity for systematic pattern recognition proved particularly valuable in mapping intervention types across diverse economic settings, enabling comprehensive identification of similar approaches that might otherwise be overlooked during manual review processes.

The artificial intelligence system's strength in comprehensive quotation extraction and terminology consistency enhanced the thoroughness of our evidence synthesis. Claude systematically identified recurring intervention characteristics and outcome measures across studies, providing a robust foundation for subsequent human analysis and interpretation. This systematic approach ensured that no potentially relevant patterns were inadvertently missed during the data processing phase, contributing to the comprehensiveness of our findings.

#### 2.5.2 Artificial intelligence limitations and validation requirements

Despite these strengths, several limitations in artificial intelligence analysis necessitate continuous human oversight and validation. Claude occasionally conflated studies from different World Bank income classifications, particularly when interventions shared similar characteristics but were implemented in different economic contexts. These classification errors required systematic human review to ensure accurate representation of how the economic setting influenced intervention selection and adaptation strategies.

The artificial intelligence system demonstrated limited capacity for assessing clinical significance and practical implementation feasibility, areas where human expertise proved indispensable. Claude's analysis focused primarily on textual patterns and explicit terminology, occasionally missing implicit cultural references and contextual nuances that significantly influenced intervention effectiveness and transferability across different healthcare settings.

#### 2.5.3 Human analytical strengths and clinical expertise

Human researchers provided essential analytical depth through critical thinking and clinical contextualisation that artificial intelligence systems cannot replicate. The capacity for thorough deliberation when resolving discrepancies between different data sources proved crucial for ensuring analytical accuracy and maintaining methodological rigour. Human expertise was particularly valuable in interpreting the clinical significance of identified patterns and assessing the practical feasibility of implementing different coping strategies across diverse resource settings.

Clinical experience enabled human researchers to recognise subtle but important distinctions between intervention approaches that appeared similar in textual description but differed significantly in implementation requirements and expected outcomes. This expertise proved essential for understanding how interventions were adapted to local contexts and for identifying factors that contributed to successful implementation across different economic settings.

#### 2.5.4 Lessons learned and practical implications

Our experience demonstrated that optimal analytical outcomes require researchers to be AI-literate rather than AI-dependent, utilising artificial intelligence capabilities whilst maintaining human oversight and interpretation. Claude's tendency to conflate World Bank regional classifications and its requirement for human guidance in clinical significance assessment illustrated the continued necessity of human expertise in systematic review processes.

The most effective approach involved leveraging artificial intelligence for systematic pattern recognition and initial data processing whilst relying on human researchers for clinical interpretation, cultural contextualisation, and practical implementation assessment. This collaborative model enhanced both the efficiency and comprehensiveness of our analysis whilst maintaining the methodological rigour essential for producing reliable and actionable findings.

Future researchers employing similar collaborative approaches should anticipate the need for systematic validation procedures to address artificial intelligence classification limitations, whilst capitalising on AI capabilities for comprehensive pattern recognition and data processing. The integration of these complementary strengths offers considerable potential for enhancing both the quality and efficiency of reviews in healthcare research, provided that human oversight remains central to the analytical process.

## 3 Results

The search strategy identified 2407 potentially relevant studies. After removing 512 duplicates, 1895 studies were screened for eligibility. After title and abstract screening, 260 studies were assessed for full-text eligibility. Of these, 185 were excluded for the following reasons:

• Wrong population (*n* = 71)
• Wrong outcomes (*n* = 86)
• Wrong study design (*n* = 24)
• Non-English language (*n* = 4)

The final review included seventy-five studies (see supplementary material 2: characteristics of the included studies) that met all the inclusion criteria. The complete selection process is detailed in the for Systematic Reviews and Preferred Reporting Items Meta-Analyses flow diagram.

Studies excluded (*n* = 183).

Wrong Population (Studies not focused on Frontline Healthcare workers (*n* = 69).

Wrong outcomes (Not addressing coping strategies for Stress management) (n = 86).

Wrong Study design (Editorials, Study Protocols and Letters to editors) (*n* = 24).

Studies other the English lnguage (*n* = 4).

### 3.1 PRISMA flow diagram



### 3.2 Research design

**Figure:**
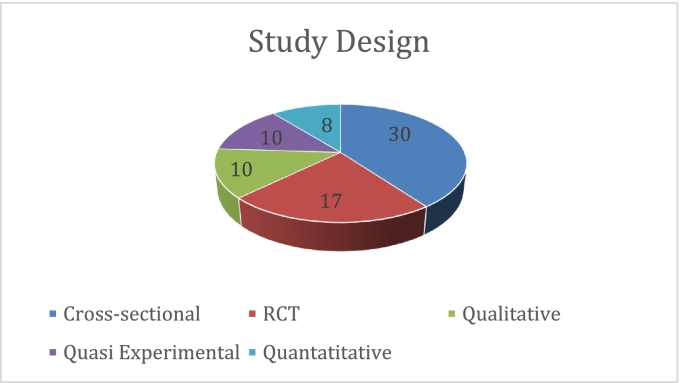

A comprehensive analysis of the research methodologies revealed the predominant use of cross-sectional designs (n = 30), followed by Randomised Controlled Trials (*n* = 17). Qualitative and quasi-experimental approaches were equally represented (*n* = 10 each), whereas quantitative studies comprised a smaller proportion (n = 8). This methodological diversity enabled a robust examination of the research objectives through various analytical lenses.

### 3.3 Study setting

The research settings encompassed multiple healthcare environments, with territorial healthcare facilities representing the majority (*n* = 32). General hospitals constituted a considerable proportion of the study population (*n* = 13), followed by secondary care facilities (n = 10). Primary care settings (n = 7) and community hospitals (n = 1) were also represented. Notably, twelve studies did not specify their healthcare settings, which warrants consideration when interpreting their findings.

### 3.4 Sample characteristics

#### 3.4.1 Sample size and distribution

The cumulative sample size across all studies was 33,889 healthcare professionals. The sample sizes varied considerably, ranging from seven participants in a Moroccan pilot study [27] examining the coaching effects on emergency nurses' stress management to 11,827 participants in a Chinese cross-sectional study [28] investigating resilience and burnout among hospital nursing staff.

#### 3.4.2 Gender distribution

The sample demonstrated a significant gender disparity, with female participants (*n* = 15,374) outnumbering male participants (*n* = 3648), reflecting the characteristic gender distribution in healthcare professions.

#### 3.4.3 Age range

Participants' ages ranged from 18 to 69 years, encompassing healthcare professionals from their early career to near retirement, providing insights across different career stages and generational perspectives.

**Figure:**
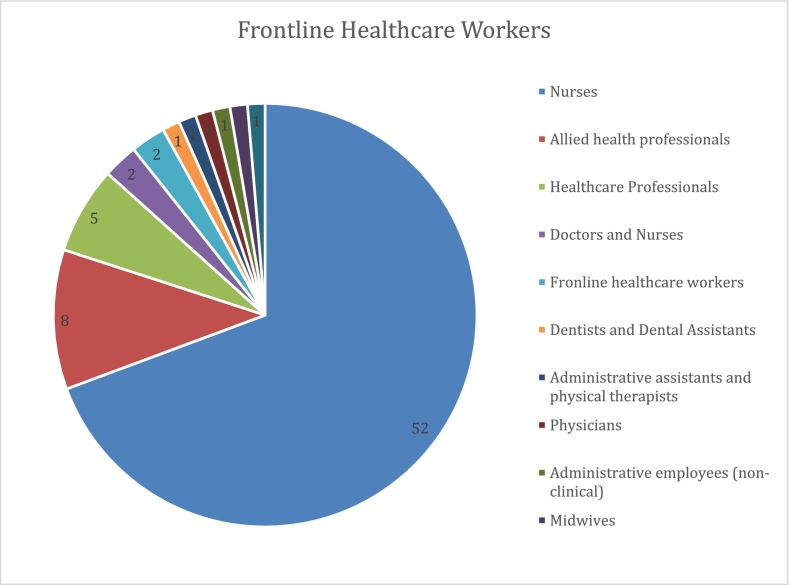

#### 3.4.4 Professional composition

The healthcare workforce representation was diverse, with nurses forming the largest professional group (*n* = 52), followed by allied health professionals (*n* = 8), and general healthcare professionals (n = 5). The sample also included doctors and nurses (*n* = 2), frontline healthcare workers (n = 2), and single representatives from various specialties including dentistry, physical therapy, midwifery, and administrative roles.

#### 3.4.5 Geographical distribution

**Figure:**
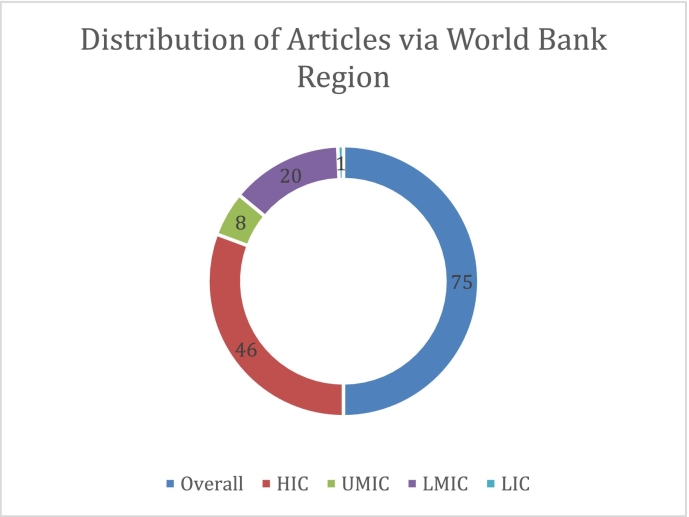

This research spanned thirty countries across multiple continents and was published from 1984 to 2024. The high-income countries have more studies (*n* = 46), followed by lower-middle-income countries (*n* = 20), upper-middle income countries (n = 8), and a single study from a low-income country. The United States contributed the largest subset (*n* = 17), followed by Iran (*n* = 9), and China (*n* = 7). Notable representations came from Italy (n = 4), while Pakistan, Spain, and Japan each contributed three studies. Several countries, including Turkey, Ghana, Brazil, India, South Korea, and the UK, conducted two studies each. The remaining countries, including Indonesia, Taiwan, Switzerland, Malawi, and Australia, each contributed one study, ensuring a globally diverse perspective in the research.

#### 3.4.6 Coping strategies

Healthcare professionals employ various coping strategies that can be categorized into several interconnected approaches. At the foundation lies social support and communication, where professionals utilize situational support mechanisms encompassing autonomy, control over practice, and group cohesion. This is enhanced through manager consideration and substantive exchange, while incorporating practical techniques such as focused breathing exercises and physical comfort like hugs. The effectiveness of these strategies is amplified through teamwork and collaborative practices, creating a supportive professional environment.

Self-management strategies form another crucial component, incorporating both proactive and reactive approaches. These include action planning, comprehensive self-care practices, and self-regulation techniques. Professionals employ both active coping and moral resilience, balancing constructive and destructive coping mechanisms while maintaining control and support strategies. This approach encompasses problem-solving and planning, effective symptom management, and the development of self-assurance and self-confidence. The implementation of adaptive strategies and appropriate delegation skills further strengthens these self-management techniques.

Cognitive-behavioral interventions and mindfulness-based approaches represent evidence-based strategies for managing professional stress. CBT techniques focus on problem-solving and positive re-appraisal, often implemented through structured models such as the Transtheoretical Coaching Model and PRECEDE-PROCEED training. Complementing these are mindfulness-based approaches, including formal programs like Mindfulness-Based Stress Reduction (MBSR) and Mindfulness-Based Cognitive Therapy (MBCT). These are supplemented by practices such as mindfulness-based yoga, Progressive Muscular Relaxation (PMR), and innovative approaches like laughter yoga.

Organizational and institutional approaches adopt a more systematic perspective, implementing comprehensive multilevel strategies that recognise multiple levels of influence. These programs integrate various elements including social support, cognitive restructuring, and desiderative thinking, often formalized through programs like Stress First Aid (SFA) and the Promoting Resilience in Nurses initiative. Creative expressions through art therapy (CAT) add another dimension to these institutional approaches, offering alternative means of stress management and emotional processing.

The integration of technology has revolutionized the delivery of coping strategies, with programs like BREATHE and WISER providing web-based support systems. These digital platforms facilitate daily and weekly web-based meetings, offer cognitive behavioral therapy modules, and even incorporate virtual chatbots combining CBT with positive psychology principles. This technological approach is complemented by organizational frameworks such as the Job demands-resource model, which addresses both health impairment and motivation processes, while considering practical aspects like night shifts and group therapy support.

**Figure:**
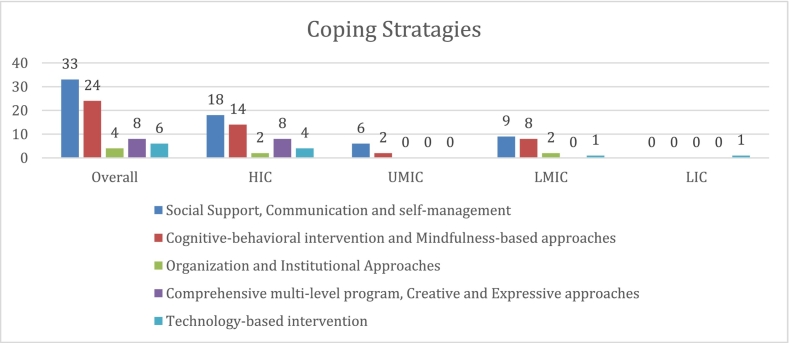

Across all income settings, organizational support and professional development play crucial roles in facilitating effective stress management. Brooks et al. (1993) found that when nurses perceived adequate situational support through autonomy, control over practice, group cohesion, and manager consideration, it led to more effective individual coping [12]. The implementation of comprehensive programs that combine individual coping strategies with institutional support mechanisms has shown the most promising results. Wallace et al. (2006) demonstrated the effectiveness of an ecological approach incorporating multiple levels of influence, with 85 % of nurses reporting improved stress handling [5]. The COVID-19 pandemic has further emphasized the importance of adaptable and resilient healthcare systems, as evidenced by McLean et al. (2023) who documented the successful adaptation of stress first aid programs, demonstrating significant improvements in peer support proficiency (73.1 % to 89.4 %) [6].

#### 3.4.7 High-income countries

In high-income countries, healthcare workers employ multiple levels of coping strategies. Individual-level approaches prominently feature mindfulness-based interventions, with studies showing that even brief 4-week mindfulness programs can significantly improve burnout symptoms and life satisfaction [11]. Cognitive-behavioral techniques, delivered through platforms like the web-based BREATHE program studied by Hersch et al. (2016), have demonstrated considerable success in stress reduction, particularly among experienced nurses [29]. Physical activity and lifestyle interventions have emerged as popular coping mechanisms, with Shechter et al. (2020) reporting that 80 % of healthcare workers actively employed coping behaviours during the COVID-19 pandemic, with physical activity (59 %), spirituality (23 %), and meditation (23 %) being the most common strategies [30].

#### 3.4.8 Upper-middle-income countries

Upper-middle-income countries exhibit distinct characteristics in their coping approaches. Religious and spiritual coping mechanisms play a central role, with Munawar (2019) highlighting how healthcare workers in Malaysia utilized religious coping as a primary strategy [31]. Innovative interventions like laughter yoga have shown promising results, with Celik (2023) demonstrating significant improvements in both psychological resilience (64.50 ± 13.17 vs 45.88 ± 11.33, *p* < 0.001) and sleep quality [32]. These countries emphasise the interplay between individual coping strategies and social support networks, as evidenced by Celestino (2020) who found that Family Health nurses in Brazil employed a combination of individual strategies (meditation, exercise, and spirituality) and social support networks [33].

#### 3.4.9 Lower-middle-income countries

Lower-middle-income countries have successfully adapted and implemented evidence-based psychological interventions. Yasmin et al. (2022) demonstrated remarkable improvements in both burnout scores (decreasing from 32.14 to 23.96, p < 0.001) and resilience measures (increasing from 25.40 to 38.16, p < 0.001) through Cognitive Behavioral Therapy among critical care nurses [34]. The implementation of mindfulness-based interventions has been particularly noteworthy, with Fatemi et al. (2024) and Talebiazar et al. (2024) showing their effectiveness in reducing occupational stress and burnout among psychiatric and geriatric nurses [35,36]. Resource-conscious implementation strategies, such as group therapy interventions studied by Jadidi et al. (2024), have proven valuable in maximizing limited resources while maintaining intervention effectiveness, showing significant reductions in job stress scores from 178.76 to 135.62 [37].

#### 3.4.10 Low-income countries

In low-income countries, innovative approaches to mental health support have emerged, particularly through digital interventions. A notable example is the implementation of a virtual chatbot (Vitalk) that integrates multiple evidence-based therapeutic approaches, including CBT and Positive Psychology. This digital intervention demonstrated significant improvements in mental health outcomes, with marked reductions in depression (−0.68), anxiety (−0.44), and burnout (−0.58), while substantially increasing resilience measures (1.47) [15].

## 4 Discussion

### 4.1 Synthesis of findings

The review reveals both common threads and distinct variations in how healthcare workers develop and maintain resilience across different economic settings. While the fundamental challenges of healthcare work stress are universal, the approaches to addressing these challenges vary significantly based on resource availability and cultural context.

Evidence suggests that effective interventions often combine multiple approaches, with mindfulness-based strategies showing promise in all economic contexts. In high-income countries, structured mindfulness programs have demonstrated significant improvement in burnout symptoms and life satisfaction [11]. These findings extend to upper-middle-income settings where innovative adaptations such as laughter yoga have shown remarkable effectiveness in improving psychological resilience [32]. Lower middle-income countries have successfully implemented mindfulness-based stress-reduction programs, with studies showing significant improvements in workplace well-being and empathy levels [35,36].

The integration of technology to deliver mental health support has emerged as a promising approach, especially in resource-constrained settings. The successful implementation of virtual chatbot intervention in Malawi, a low-income country, demonstrates how digital solutions can effectively support resilience among healthcare workers [15]. This aligns with the findings from other settings, such as Vietnam's successful smartphone-based intervention [38] and web-based programs in high-income countries [29].

Cultural and contextual adaptations play a crucial role in successful interventions. Religious and spiritual coping mechanisms are prominent in upper-middle-income country studies [31,39], while high-income country research tends to emphasise individual psychological approaches [5,12]. This underscores the importance of considering cultural frameworks when developing resilience interventions.

Organizational support has emerged as a critical factor in all economic settings. Studies in high-income countries emphasise the importance of structural support and professional development [7,40]lower-resource innovative approaches to maximise limited institutional resources [33,37]. The relationship between professional identity and resilience appears particularly strong, with studies across different settings showing significant correlations between resilience and professional commitment [41].

The COVID-19 pandemic has provided unique insights into healthcare workers' resilience in different economic contexts. Studies from various settings have demonstrated how healthcare workers adapted their coping strategies during crises, with physical activity and mindfulness practices emerging as common approaches [6,30]. The pandemic also saw a rapid evolution in support systems, with many institutions developing innovative approaches to maintain healthcare worker resilience.

A notable finding was the effectiveness of problem-focused coping strategies in different economic settings. Studies from high-income countries [42], upper-middle-income countries [43], and lower-middle-income countries [44] have consistently shown the value of practical, solution-oriented approaches to stress management.

The role of professional experience in developing resilience appears to be consistent across settings, with multiple studies indicating that longer professional experiences correlate with higher resilience levels [3]. This suggests the importance of considering career stage when developing support interventions.

### 4.2 Alignment with WHO global health priorities and SDGs

#### 4.2.1 WHO global health priorities

This research directly supports the World Health Organisation's 13th General Programme of Work (GPW 13) 2019–2025, particularly in achieving universal health coverage through sustainable healthcare workforce development [45]. Healthcare worker resilience represents a fundamental prerequisite for sustainable health service delivery, with our findings demonstrating how evidence-based stress management interventions can reduce turnover rates and improve care quality, directly supporting universal health coverage objectives.

The COVID-19 pandemic findings within our review align closely with the WHO's health emergency preparedness framework, emphasising the critical importance of psychological support systems during health crises [46]. Our analysis of pandemic-specific interventions provides evidence-based guidance for maintaining healthcare workforce capacity during health emergencies, supporting the WHO's strategic priority of addressing health emergencies through resilient health systems.

Our findings support the WHO's Framework on Integrated People-Centred Health Services, particularly the workforce development strategy emphasising “empowering and engaging health workers” [47]. The evidence for workplace well-being interventions that can be integrated into health system strengthening efforts directly advances WHO's quality improvement frameworks through documented relationships between healthcare worker resilience and patient safety outcomes.

#### 4.2.2 Sustainable development goals alignment

The review contributes to Sustainable Development Goal 3 (Ensure healthy lives and promote well-being for all), particularly Target 3.4 (promote mental health and well-being) and Target 3.8 (achieve universal health coverage) [4]. Healthcare worker well-being emerges as essential for achieving these targets, with our findings demonstrating the relationship between worker resilience and care quality across diverse economic settings.

## 5 Conclusion

This comprehensive review demonstrates that, while healthcare workers across different economic settings face similar challenges, the approaches to building and maintaining resilience must be tailored to specific contexts while incorporating universal effective elements. The success of various interventions, from traditional mindfulness approaches to innovative digital solutions, suggests that effective resilience building is possible even in resource-constrained environments.

These findings highlight several key areas for future research. First, interventions should combine multiple approaches that incorporate both individual and organizational elements. Second, cultural sensitivity and contextual adaptation are crucial for successful intervention. Third, this technology offers promising solutions for scaling support, particularly in resource-limited settings. Fourth, professional development and support systems play vital roles in building sustainable resilience.

Future research should focus on developing and evaluating sustainable and scalable interventions that can effectively support healthcare workers working within resource constraints. Particular attention should be paid to the long-term effectiveness of different intervention types, role of technology in delivering support, and integration of resilience building into professional education and training.

The COVID-19 pandemic has highlighted both challenges and opportunities to support healthcare worker resilience. As global healthcare continues to face unprecedented challenges, insights from this review will provide valuable guidance for developing effective support systems across different economic settings. The success of various interventions demonstrates that with appropriate adaptation and implementation, healthcare worker resilience can be effectively supported regardless of resource constraints.

### 5.1 Review limitations

Several methodological limitations must be acknowledged when interpreting these findings. The predominance of cross-sectional study designs (*n* = 30, 40 %) limits our ability to establish causal relationships between coping strategies and resilience. Most included studies assessed immediate post-intervention outcomes rather than conducting prospective evaluations of long-term retention and application of coping strategies in practice [28]. This represents a significant gap in understanding the sustainability of the intervention's effects over time.

The heterogeneity in outcome measures across studies presented challenges for synthesis, with diverse instruments used to assess stress, resilience, and coping. Additionally, the exclusion of non-English studies may have introduced cultural bias, particularly affecting representation of indigenous and traditional coping practices that could inform culturally-adapted interventions [23].

Sample characteristics revealed significant sex disparity, with female participants substantially outnumbering males (*n* = 15,374 vs. *n* = 3648), reflecting healthcare workforce demographics but potentially limiting generalisability to male healthcare workers. Economic setting representation was similarly unbalanced, with high-income countries contributing disproportionately more studies (*n* = 46, 61 %), potentially skewing intervention recommendations towards resource-intensive approaches.

### 5.2 Future research recommendations

Based on this comprehensive scoping review's findings, future research should prioritise conducting rigorous randomised controlled trials in lower-middle-income countries (LMIC) that specifically evaluate technology-based interventions combining cognitive-behavioral therapy principles with culturally adapted elements. The review demonstrates a significant research gap in LMIC settings, where only 20 of the 75 included studies were conducted, yet these contexts showed remarkable success with resource-conscious interventions such as the virtual chatbot (Vitalk) in Malawi that achieved substantial improvements in mental health outcomes (depression −0.68, anxiety −0.44, burnout −0.58, resilience +1.47) [15]. Given that cognitive-behavioral interventions proved effective across all economic settings, with studies in LMIC showing significant improvements in both burnout and resilience scores through CBT-based approaches, future RCTs should focus on developing and testing scalable digital platforms that integrate evidence-based CBT techniques while incorporating locally relevant cultural frameworks, including religious and spiritual coping mechanisms that emerged as prominent features in upper-middle and lower-middle-income country studies. Such research would address the critical need for cost-effective, culturally sensitive, and technologically accessible mental health support systems that can be sustainably implemented across diverse LMIC healthcare settings, ultimately contributing to global health equity in healthcare worker wellbeing support.

### 5.3 Policy recommendations

The synthesis suggests several key policy recommendations for healthcare organisations.

1. Integration of Regular Resilience Training Programs into a Professional Development Framework.
2. Establishment of accessible support systems that combine in-person and digital resources.
3. Development of tailored interventions considering experience levels and professional roles.
4. Creation of supportive organizational cultures that recognise and address workplace stressors.
5. Integration of culturally appropriate stress management techniques, including religious and spiritual coping mechanisms.
6. Development of cost-effective interventions suitable for resource-limited settings.
7. Strengthening of social support networks within healthcare organisations.
8. Implementation of professional development programs that enhance both technical and coping skills.

## Funding

This review was supported by the 10.13039/501100010221Higher Education Commission of Pakistan through the National Research Program for Universities (HEC-NRPU-16055).

## Availability of data, code, and other materials

All data extraction forms, data extracted from the included studies, and data used for analyses are available from the corresponding author upon reasonable request.

## CRediT authorship contribution statement

**Bilal Ahmad:** Writing – original draft, Project administration, Methodology, Formal analysis, Data curation, Conceptualization. **Khalid Rehman:** Writing – review & editing, Supervision, Project administration, Conceptualization. **Shawana Bangash:** Software, Methodology. **Rida Zarkaish:** Software, Methodology. **Mahdia Babak:** Software, Methodology. **Sana Rehman:** Software, Methodology. **Samina Ihsan:** Software, Methodology. **Farhad Ali Khattak:** Writing – original draft, Formal analysis. **Muhammad Irfan:** Writing – original draft, Conceptualization. **Asif Rehman:** Writing – original draft. **Abdul Jalil Khan:** Writing – original draft, Conceptualization. **Zohaib Khan:** Writing – original draft, Supervision, Conceptualization.

## Declaration of competing interest

The authors declare no conflicts of interest.

## Appendix A Supplementary data

Supplementary material 1 Image 2

Supplementary material 2 Image 3
