# Supplementary material for: Coping strategies and resilient behavior among frontline healthcare workers: A scoping review
Source: Dialogues Health. 2025 Oct 23;7:100252. doi: 10.1016/j.dialog.2025.100252 (PMC12664065; doi:10.1016/j.dialog.2025.100252)
Supplement: Supplementary file 1 — Supplementary material 1 [file mmc1.docx]

**Supplementary material 1: Search Strategy**

**Database: APA PsycInfo <1806 to November 2024 Week 4>, Embase <1974 to 2024 November 27>, Ovid MEDLINE(R) ALL <1946 to November 27, 2024>**
**Search Strategy:**
**1**  Frontline healthcare work*.mp. [mp=ti, ab, hw, tc, id, ot, tm, mf, tn, dm, dv, kf, fx, dq, bt, nm, ox, px, rx, an, ui, sy, ux, mx] (1571)
**2**  Healthcare work*.mp. [mp=ti, ab, hw, tc, id, ot, tm, mf, tn, dm, dv, kf, fx, dq, bt, nm, ox, px, rx, an, ui, sy, ux, mx] (67422)
**3**  allied health*.mp. [mp=ti, ab, hw, tc, id, ot, tm, mf, tn, dm, dv, kf, fx, dq, bt, nm, ox, px, rx, an, ui, sy, ux, mx] (50924)
**4**  nurse*.mp. [mp=ti, ab, hw, tc, id, ot, tm, mf, tn, dm, dv, kf, fx, dq, bt, nm, ox, px, rx, an, ui, sy, ux, mx] (1034693)
**5**  medical doctor.mp. [mp=ti, ab, hw, tc, id, ot, tm, mf, tn, dm, dv, kf, fx, dq, bt, nm, ox, px, rx, an, ui, sy, ux, mx] (4753)
**6**  1 or 2 or 3 or 4 or 5 (1133785)
**7**  Stress manag*.mp. [mp=ti, ab, hw, tc, id, ot, tm, mf, tn, dm, dv, kf, fx, dq, bt, nm, ox, px, rx, an, ui, sy, ux, mx] (31390)
**8**  psycholog* resilient.mp. [mp=ti, ab, hw, tc, id, ot, tm, mf, tn, dm, dv, kf, fx, dq, bt, nm, ox, px, rx, an, ui, sy, ux, mx] (129)
**9**  psycholog* resilien*.mp. [mp=ti, ab, hw, tc, id, ot, tm, mf, tn, dm, dv, kf, fx, dq, bt, nm, ox, px, rx, an, ui, sy, ux, mx] (17516)
**10**  Coping Stratag*.mp. [mp=ti, ab, hw, tc, id, ot, tm, mf, tn, dm, dv, kf, fx, dq, bt, nm, ox, px, rx, an, ui, sy, ux, mx] (4)
**11**  7 or 8 or 9 or 10 (48636)
**12**  Hospital.mp. [mp=ti, ab, hw, tc, id, ot, tm, mf, tn, dm, dv, kf, fx, dq, bt, nm, ox, px, rx, an, ui, sy, ux, mx] (4819020)
**13**  healthcare facilit*.mp. [mp=ti, ab, hw, tc, id, ot, tm, mf, tn, dm, dv, kf, fx, dq, bt, nm, ox, px, rx, an, ui, sy, ux, mx] (25989)
**14**  healthcare setting.mp. [mp=ti, ab, hw, tc, id, ot, tm, mf, tn, dm, dv, kf, fx, dq, bt, nm, ox, px, rx, an, ui, sy, ux, mx] (10649)
**15**  Primary care.mp. [mp=ti, ab, hw, tc, id, ot, tm, mf, tn, dm, dv, kf, fx, dq, bt, nm, ox, px, rx, an, ui, sy, ux, mx] (416810)
**16**  Secondary care.mp. [mp=ti, ab, hw, tc, id, ot, tm, mf, tn, dm, dv, kf, fx, dq, bt, nm, ox, px, rx, an, ui, sy, ux, mx] (30190)
**17**  Tertiary care.mp. [mp=ti, ab, hw, tc, id, ot, tm, mf, tn, dm, dv, kf, fx, dq, bt, nm, ox, px, rx, an, ui, sy, ux, mx] (295808)
**18**  12 or 13 or 14 or 15 or 16 or 17 (5312791)
**19**  6 and 11 and 18 (1139)
