# Supplementary material for: Coping strategies and resilient behavior among frontline healthcare workers: A scoping review
Source: Dialogues Health. 2025 Oct 23;7:100252. doi: 10.1016/j.dialog.2025.100252 (PMC12664065; doi:10.1016/j.dialog.2025.100252)
Supplement: Supplementary file 2 — Supplementary material 2 [file mmc2.docx]

**Supplementary material 2: Characteristics of the included studies**

| **Study ID** | **Study Design** | **Country** | **Age** | **Sample Size** | **Setting** | **Frontline Healthcare worker** | **Coping Strategy** | **Category of Coping Strategy** | **Resilient Behavior** | **Key Finding (30 Words)** |
| --- | --- | --- | --- | --- | --- | --- | --- | --- | --- | --- |
| **HIGH INCOME COUNTRIES** | | | | | | | | | | |
| Brooks,1993^1^ | Quantitative | USA^HIC^ | NR | NR | General Hospital | Nurses | Situational Support (which includes autonomy, control over practice, group cohesion, manager consideration, and substantive exchange) | Social Support, Communication, and self-management | Group Cohesion (feeling integrated into work groups and organization) | When nurses perceive situational support mechanisms through autonomy, control, group cohesion, manager consideration and substantive exchange, it leads to effective individual coping and preserves personal/professional integrity, improving patient care quality. |
| Begley,1998^2^ | Quantitative | USA^HIC^ | NR | NR | General Hospital | Allied health professionals | Action Planning | Social Support, Communication, and self-management | Acceptance | Action planning and seeking emotional support decreased during consolidation while intent to quite increased. Acceptance was effective in reducing turnover, but alcohol/drug use predicted higher distress and somatic complaints. |
| Mackenzie 2006^3^ | RCT | USA^HIC^ | 25 - 60 | N=57 (M=4, F=53) | General Hospital | Nurses | Mindfulness-based stress reduction (MBSR) | Cognitive-behavioral intervention and Mindfulness-based approaches | Present-moment awareness with nonjudgmental acceptance | The 4-week mindfulness intervention led to significant improvements in burnout symptoms, relaxation, and life satisfaction compared to controls. Mindfulness training appears effective for nurses' stress management even in brief format. |
| Wallace, 2006^4^ | Quantitative | USA^HIC^ | NR | N=275 (F=275) | General Hospital | Nurses | Ecological approach incorporating multiple levels of influence - intrapersonal (health education), interpersonal (group discussions), institutional (worksite programs), community and policy levels | Comprehensive multi-level program, Creative and Expressive approaches | Active participation in stress management workshop with relaxation techniques (diaphragmatic breathing and yoga) | Using an ecological approach, 85% of nurses reported improved stress handling and 97% found the workshop professionally valuable. Multi-level interventions including education, institutional support and relaxation techniques were effective. |
| Hersch, 2016^5^ | RCT | USA^HIC^ | 22 - 65 | N=104 (M=13, F=131) | Not defined | Nurses | Web-based BREATHE program | Technology-based intervention | Cognitive-behavioral techniques | The web-based BREATHE program significantly reduced nurses' stress through cognitive-behavioral techniques. More experienced nurses benefited more. Program focused on changing views of stressors and stress response management. |
| Jordan, 2016^6^ | Cross-Sectional | USA^HIC^ | 41 (10.8) years | N=120 (M=4, F=116) | Territory | Nurses | Combined approach of talking to friends/loved ones, music, meditation, and eating behaviors | Comprehensive multi-level program, Creative and Expressive approaches | Situation-specific coping confidence and self-efficacy development | Nurses in high stress/poor coping group had poorest health outcomes. The combined influence of perceived stress and coping adequacy significantly impacted health behaviors and work performance. Programs should equally focus on stress reduction and coping skills. |
| Magtibay, 2017^7^ | Quasi Experimental Study | USA^HIC^ | 24 - 63 | N=50 (M=4, F=46) | Territory | Nurses | SMART (Stress Management and Resiliency Training) | Cognitive-behavioral intervention and Mindfulness-based approaches | Mindfulness | Blended learning SMART program showed significant decreases in anxiety (45.2%), stress (29.8%), and burnout (33.6%), while increasing resilience, happiness, and mindfulness among nurses at 8- and 24-weeks follow-up. |
| Mealer, 2017^8^ | Qualitative | USA^HIC^ | NR | N=33 (M=1, F=32) | Territory | Nurses | Mindfulness-based cognitive therapy (MBCT) | Cognitive-behavioral intervention and Mindfulness-based approaches | Recovery, sustainability, and growth approach | Focus groups identified barriers and incentives for MBCT implementation. Key recommendations included hybrid delivery format, ICU-specific content, dual instructors (psychotherapist and ICU nurse), and short mindfulness practices. |
| Blackburn, 2020^9^ | Quasi Experimental Study | USA^HIC^ | NR | N=164 | Territory | Administrative assistants and physical therapists | Self-care | Social Support, Communication, and self-management | Mindfulness | The THRIVE program increased resilience scores (72 to 85) and decreased burnout (41 to 23) and secondary trauma (32 to 19) among oncology nurses through mindfulness, art therapy, and self-care strategies. |
| Dutton, 2020^10^ | Quasi Experimental Study | USA^HIC^ | 30 - 49 years | N=31 (M=2, F=29) | Not defined | Nurses | Web-based BREATHE program | Technology-based intervention | Mindfulness | The BREATHE web-based stress management program significantly reduced nurses' stress scores, particularly in areas of death/dying, physician conflict, workload, and treatment uncertainty (p<0.05). Program satisfaction was high. |
| Hilcove, 2020^11^ | RCT | USA^HIC^ | 24 - 69 | N=80 (M=4, F=76) | Territory | Allied health professionals | Yoga (specifically mindfulness-based yoga) | Cognitive-behavioral intervention and Mindfulness-based approaches | Mindfulness | Mindfulness-based yoga significantly improved nurses' stress levels, burnout, vitality, sleep quality, serenity, and mindfulness compared to control group. The 6-week intervention demonstrated effectiveness as a self-care modality for managing stress. |
| Shechter, 2020^12^ | Cross-Sectional | USA^HIC^ | 25 - 34 years | N=657 (M=191, F=466) | Primary | Nurses | Physical exercise/activity | Social Support, Communication, and self-management | Mindfulness | Eighty percent of healthcare workers used coping behaviors during COVID-19, with physical activity (59%), spirituality (23%), and meditation (23%) being most common. These helped reduce psychological distress and burnout. |
| Croghan, 2021^13^ | Cross-Sectional | USA^HIC^ | 30 - 60 | N=302 (M=44, F=252) | Territory | Allied health professionals | Communication (Daily and weekly web-based meetings to help manage stress) | Technology-based intervention | Bouncing-back (To bounce back from adversity and view adversity as an opportunity for growth) | Older age was associated with higher resilience. |
| Profit, 2021^14^ | RCT | USA^HIC^ | NR | N=481 (M=81, F=400) | Territory | Allied health professionals | WISER (Web-based Implementation for Science of Enhancing Resilience) | Technology-based intervention | Mindfulness | WISER intervention significantly reduced burnout, improved work-life integration, and depression among healthcare workers at 1-month and 6-months post-intervention. The intervention used positive psychology principles delivered via mobile platform. |
| McLean, 2023^15^ | Cross-Sectional | USA^HIC^ | NR | N=246 | Primary | Nurses | Stress First Aid (SFA) | Comprehensive multi-level program, Creative and Expressive approaches | Peer support | Program was rated highly acceptable and feasible by participants. Pre-post improvements in proficiency supporting peers (73.1% to 89.4%, p=0.002). Program provided shared language for discussing stress, normalized stress reactions, helped staff feel valued and connected. |
| Torres, 2023^16^ | RCT | USA^HIC^ | 28 - 60 | N=20 (M=2, F=18) | Primary | Allied health professionals | Creative Arts Therapy (CAT) | Comprehensive multi-level program, Creative and Expressive approaches | Group Expression | CAT program supported healing and resilience through building community via four key components: 1) Diversity of participants' disciplines and roles, 2) Physical separation from workplace, 3) Skilled CAT facilitators, 4) Being pushed out of comfort zones through creative activities. Program helped healthcare workers feel less isolated and more connected during pandemic stress. |
| Sos, 2024^17^ | Qualitative | USA^HIC^ | 47.63 (13.66) years | N=48 | Not defined | Healthcare Professionals | Focused breathing exercises and caring touch (hug) | Social Support, Communication, and self-management | Mindfulness techniques (Deep Breathing) | Participants reported benefits on mindfulness interventions, with 15-minute sessions being optimal. |
| Lin, 2019^18^ | RCT | China^HIC^ | 32.86 years | N=90 (M=6, F=84) | Territory | Nurses | Mindfulness-based stress reduction (MBSR) | Cognitive-behavioral intervention and Mindfulness-based approaches | Nonjudgmental awareness and acceptance | Modified MBSR program significantly reduced perceived stress and negative affect while increasing positive affect and resilience. Program showed potential to improve job satisfaction among Chinese hospital nurses. |
| Huang, 2021^19^ | Qualitative | China^HIC^ | 29.78 (5.23) years | N=23 | Not defined | Nurses | Self-regulation | Social Support, Communication, and self-management | Mental resilience transformation (from negative to positive mental state) | Nurses displayed ability to transform from negative emotional states to positive strengths through self-regulation, social support, professional achievement, infectious disease protocol familiarity, and trust in infection control measures. |
| Li , 2021^20^ | Cross-Sectional | China^HIC^ | 33.5 (9.5) years | N=309 (M=8, F=301) | Territory | Doctors and nurses | Active coping | Social Support, Communication, and self-management | Adaptation | Active coping and resilience significantly reduced COVID-19 stress among healthcare workers. Higher resilience (r=0.61) and active coping strategies were associated with better stress management and improved quality of life. |
| Jiang, 2022^21^ | Qualitative | China^HIC^ | 23 - 46 | N=17 (M=6, F=11) | Territory | Nurses | Self-management | Social Support, Communication, and self-management | Altruism-motivated coping | The study identified three main themes affecting nurse resilience during COVID-19: (1) Risk factors impacting resilience like heavy workload and stress, (2) Promoting factors including management support and social support, and (3) Motivation through altruism and sense of mission. Success in maintaining resilience required balancing these factors through both organizational support and individual coping strategies. |
| Chen, 2024^22^ | Cross-Sectional | China^HIC^ | 19 - 53 | N=569 (M=32, F=537) | Territory | Nurses | Moral Resilience | Social Support, Communication, and self-management | Self-preservation | Four moral resilience profiles identified: high (28.7%), moderate (52.3%), low responses/high efficacy (16.2%), and low (2.8%). Higher education and ethics training predicted better moral resilience and reduced compassion fatigue. |
| Sun, 2024^23^ | Quantitative | China^HIC^ | 21 - 47 | N=339 (M=21, F=318) | Territory | Nurses | Positive coping | Social Support, Communication, and self-management | Social Support | Social support had positive effects on coping strategies and negative effects on perceived stress and mental health. Path analysis showed social support moderated the relationship between perceived stress and mental health in frontline nurses. |
| Zhang, 2024^24^ | Cross-Sectional | China^HIC^ | 20 - 30 | N=11827 | Primary | Nurses | Job demands-resource model approach combining health impairment and motivation processes | Organization and Institutional Approaches | - Tenacity - Optimism   Strength (As measured by Connor-Davidson resilience scale) | Resilience mediates burnout to well-being relationship (16.91% effect). Fifteen variables predict 57.80% variance in well-being, including individual factors (age, education, health) and organizational factors (training, income, shifts). |
| Rinaldi , 2019^25^ | Quasi Experimental Study | Italy^HIC^ | 31 - 56 | N=20 (M=5, F=15) | Territory | Physicians | Mindfulness-based approach | Cognitive-behavioral intervention and Mindfulness-based approaches | Self-empathy | The Focusing intervention significantly reduced perceived stress (p=0.01) among healthcare workers, improving emotional self-regulation, mental clarity, coping abilities and self-awareness through body-oriented mindfulness techniques and self-empathy development. |
| Collantoni, 2021^26^ | Cross-Sectional | Italy^HIC^ | 42.1 years | N=996 (M=241, F=755) | Territory | Allied health professionals | Teamwork | Social Support, Communication, and self-management | Social Support | Healthcare workers in COVID-19 units showed greater coping abilities than others. Physicians demonstrated superior coping skills versus nurses. Being female and nursing role were associated with higher psychological vulnerability and lower resilience. |
| Guerra, 2022^27^ | RCT | Italy^HIC^ | 36.8 years | N=33 (M=18, F=15) | Territory | Dentists and Dental Assistants | Yoga | Cognitive-behavioral intervention and Mindfulness-based approaches | Meditation | The yoga intervention significantly improved mental composite scores (MCS) from pre- to post-intervention (p=0.002) and between intervention vs control groups (p=0.006). The 15-minute workplace yoga sessions were deemed practical and accessible for healthcare professionals. |
| Vallone, 2024^28^ | Cross-Sectional | Italy^HIC^ | 21 - 65 | N=265 (M=105, F=160) | Territory | Nurses | Active coping (specifically problem-focused coping) | Social Support, Communication, and self-management | Solution-oriented (identified in the Active/Solution-Oriented pattern group) | Study identified three distinct coping patterns among nurses: Active/Solution-Oriented (33.1%), Dysregulated/Emotion-focused (21.9%), and Passive/Disengaged (46%). Active/Solution-Oriented group showed better mental health outcomes and lower stress levels compared to other groups. |
| Martinez-Zaragoza, 2020^29^ | Cross-Sectional | Spain^HIC^ | 40.22 (8.50) years | N=96 (M=10, F=90) | Secondary | Nurses | Problem-focused coping | Social Support, Communication, and self-management | Planning | Problem-focused coping was most used (45.25%) during direct care and medication tasks. Emotion-focused coping (44%) was used broadly except in medication/documentation. Mood significantly influenced coping strategy selection. |
| Sánchez-Zaballos, 2020^30^ | Cross-Sectional | Spain^HIC^ | 43.5 years | N=320 (M=58, F=262) | Not defined | Allied health professionals | Night shifts (The study found working night shifts was associated with higher resilience, suggesting night shift work may help develop better coping abilities) | Organization and Institutional Approaches | Experience (The study found longer professional experience increased probability of having high resilience levels - OR=0.906, p=0.02) | Higher resilience was associated with being a physician, working night shifts, and having more work experience. Being single/divorced, being a nurse, and working day shifts predicted lower resilience levels. |
| Buch-Vicente, 2022^31^ | cross sectional | Spain^HIC^ | 35 - 50 | N=269 | General Hospital | Frontline healthcare workers | Problem-solving, emotional expression, social support, cognitive restructuring, desiderative thinking | Comprehensive multi-level program, Creative and Expressive approaches | Problem-focused coping | The most used strategies were problem-solving, desiderative thinking and social support. Women used more emotional expression and social support. Age and work unit risk level influenced coping strategy selection. |
| Chen, 2009^32^ | Cross-Sectional | Japan^HIC^ | 25 - 40 | N=121 | Not defined | Nurses | Constructive and Destructive coping strategies | Social Support, Communication, and self-management | Self-esteem | Professional status, patient safety, and OR environment correlated positively with constructive coping strategies. Job satisfaction factors like work rewards, OR environment, and administrative management correlated negatively with destructive coping strategies. |
| Lee, 2011^33^ | Cross-Sectional | Japan^HIC^ | 20 - 59 | N=1144, M=34, F=1110) | General Hospital | Nurses | Control and Support strategies | Social Support, Communication, and self-management | Self-actualization | Work stress and stress-coping strategies were significantly positively correlated. Nurses with high work stress showed better coping strategies. |
| Watanabe, 2019^34^ | RCT | Japan^HIC^ | 21–55 | N=80 (F=80) | Secondary | Nurses | Brief mindfulness-based stress management program | Cognitive-behavioral intervention and Mindfulness-based approaches | Cognitive behavioral regulation | No significant difference between mindfulness program and leaflet groups in mental state outcomes. Program provided by senior nurses showed neither benefits nor harm in maintaining mental health state. |
| Ibrahim , 2019^35^ | Cross-Sectional | Brunei^HIC^ | **20 - 50** | N=85 (M=26, F=59) | Territory | Nurses | Problem-solving and positive re-appraisal | Cognitive-behavioral intervention and Mindfulness-based approaches | Planful problem-solving approach | Nurses primarily used problem-oriented coping strategies, particularly planful problem-solving and positive re-appraisal. |
| Betke, 2021^36^ | Cross-Sectional | Poland^HIC^ | 22 - 52 years | N=91 (M=1, F=90) | Not defined | Nurses | Active coping and planning | Social Support, Communication, and self-management | Sense of coherence | Nurses with higher sense of coherence used more adaptive coping strategies and less avoidance strategies. They showed better mental health and functioning in stressful work environments through active problem-focused coping approaches. |
| Klemets, 2017^37^ | Qualitative | Norway^HIC^ | NR | NR | Territory | Nurses | Collaboration | Social Support, Communication, and self-management | Adaptation | Nurses use four main collaborative strategies to manage nurse call interruptions: formally sharing responsibilities, informally sharing responsibilities, staying connected, and discerning call urgency to maintain work efficiency and patient safety. |
| Dumarkaite, 2023^38^ | RCT | Lithuania^HIC^ | NR | N=209 | Primary | Nurses | CBT-based (Cognitive Behavioral Therapy) | Cognitive-behavioral intervention and Mindfulness-based approaches | Recovery | The CBT-based behavioral therapy improved stress recovery skills (psychological detachment, relaxation, mastery), reduced stress/anxiety/depression symptoms, and increased psychological well-being with effects stable at 3-month follow-up. High user satisfaction (>80%) reported. |
| Foster, 2024^39^ | RCT | Australia^HIC^ | 35.4 years | N=154 (M=39, F=104) | Territory | Nurses | Resilience Programme (Promoting Resilience in Nurses) | Comprehensive multi-level program, Creative and Expressive approaches | Self-Efficacy | Intervention significantly improved mental health nurses' coping self-efficacy, reduced psychological distress, enhanced wellbeing, resilience, and posttraumatic growth over three months. |
| Ma, 2024^40^ | Qualitative | Taiwan^HIC^ | 30 - 50 | N=8 (M=5, F=3) | Territory | Doctors and Nurses | Mindfulness-Based Stress reduction | Cognitive-behavioral intervention and Mindfulness-based approaches | 1. Calm personality 2. Optimistic attitude 3. Communication   Overcoming challenges | Healthcare workers experienced significant stress from COVID-19 exposure, heavy workloads, and lack of resources. Mindfulness-based stress reduction and resilience helped maintain mental health through optimistic attitudes, rational communication, and support systems. |
| Uccella, 2024^41^ | Cross-Sectional | Switzerland^HIC^ | 18 - 65 | N=1197 (M=355, F=842) | Territory | Healthcare Professionals | 1. Physical activity 2. Mindfulness 3. Religious 4. Social Support 5. Meditation practices | Comprehensive multi-level program, Creative and Expressive approaches | 1. Good collaboration  2. Optimistic Attitude  3. Work-life balance | Male sex, older age, seniority, higher rank, physical activity, mindfulness practices, religiosity, and good workplace collaboration were associated with higher resilience among hospital workers. Physicians and hospitality staff showed highest resilience levels, particularly in surgery and emergency departments. |
| Tyler,1992^42^ | Quantitative | UK^HIC^ | NR | N=72 | General Hospital | Nurses | Active Cognitive and Active Behavioral Coping | Cognitive-behavioral intervention and Mindfulness-based approaches | Social Support | Active coping strategies like seeking help and reorganizing work were more effective than avoidance coping. Poor mental health outcomes were predicted by excessive workload and use of avoidance coping strategies. |
| Bruneau, 2004^43^ | Qualitative | UK^HIC^ | NR | N=18 | Community Hospital | Nurses | Progressive Muscular Relaxation (PMR) | Cognitive-behavioral intervention and Mindfulness-based approaches | Detachment | Most nurses utilized family/home support rather than workplace support. They employed detachment and systematic analytical approaches. A stress-reduction program teaching PMR showed limited effectiveness, with only 33% practicing the technique. |
| Rosenthal,1989^44^ | Quantitative | Maryland^HIC^ | 20 - 30 | N=30 (F=30) | General Hospital | Nurses | Problem-Oriented and Appraisal-Oriented Strategies (including logical analysis, information seeking, and problem solving) | Comprehensive multi-level program, Creative and Expressive approaches | Affective Regulation (including seeking emotional support, getting busy with other activities, and seeing the positive side of situations) | Nurses used various problem-oriented and emotion-oriented coping strategies they found helpful. Problem-focused strategies were most common and effective, while avoidance/emotional discharge strategies were least effective in managing NICU stress. |
| Baeyer,1984^45^ | RCT | Canada^HIC^ | NR | N=14 | General Hospital | Nurses | Cognitive-behavioral stress management training | Cognitive-behavioral intervention and Mindfulness-based approaches | Self-monitoring | Cognitive-behavioral stress management training was effective in reducing anxiety among inexperienced nurses but not experienced nurses. Benefits appeared linked to increased understanding of stress rather than behavioral changes. |
| Dabou, 2022^46^ | Cross-Sectional | United Arab Emirates^HIC^ | 38.81 (7.23) years | N=64 (M=3, F=61) | Secondary | Nurses | Delegation | Social Support, Communication, and self-management | Developing resilience | The study found that effective delegation and developing resilience were the most helpful interventions. A significant positive correlation existed between four stress management elements: eliminating stressors, developing resiliency, short-term coping, and effective delegating (p=0.00). |
| **Upper-Middle Income Countries** | | | | | | | | | | |
| Munawar, 2019^47^ | Qualitative study | Malaysia^UMIC^ | 28 - 38 | N=15 (M=15) | General Hospital | Frontline emergency healthcare workers | Religious coping | Social Support, Communication, and self-management | Problem-focused | Healthcare workers utilized religious coping, limiting media exposure, and viewing pandemic as "just another emergency". Self-control and positive attitudes served as buffers against stress while maintaining professional responsibilities. |
| Oksuz, 2018^48^ | Cross-Sectional | Turkey^UMIC^ | 36 - 40 years | N=242 (M=18, F=224) | Territory | Nurses | Resilience Behavior | Social Support, Communication, and self-management | social support and job satisfaction | The resilience of the nurses was moderate, and the nurses were observed to be competent in maintaining, planning, and organising their daily work; to have good self‐confidence, positive plans for the future, and good family relationships and family support, and to be eager to participate in social activities. |
| Celik, 2023^49^ | RCT | Turkey^UMIC^ | 29.15 (7.06) years | N=90 (M=12, F=78) | Secondary | Nurses | Laughter Yoga | Cognitive-behavioral intervention and Mindfulness-based approaches | Deep breathing | Laughter yoga significantly improved psychological resilience (64.50 ± 13.17 vs 45.88 ± 11.33, p<0.001) and sleep quality (10.06 ± 3.02 vs 16.93 ± 2.53, p<0.001) in nurses compared to control group after 8 sessions over 4 weeks. |
| Umann, 2014^50^ | Cross-Sectional | Brazil^UMIC^ | 20 - 59 | N=151 (M=14, F=137) | Not defined | Nurses | Symptoms management | Social Support, Communication, and self-management | Control strategies | Symptoms management strategy had a positive effect in reducing nurses' stress. Control and avoidance strategies increased stress levels. Experience level and institutional time influenced coping effectiveness among hemato-oncology nurses. |
| Celestino, 2020^51^ | Qualitative | Brazil^UMIC^ | 36 - 40 years | N=18 (M=2, F=16) | Primary | Nurses | Individual strategies (meditation, exercise, spirituality) | Cognitive-behavioral intervention and Mindfulness-based approaches | Social support networks | Family Health nurses use individual coping strategies like physical activity, meditation, spirituality and seeking family support to manage work-related psychosocial risks due to insufficient resources and high workload. |
| Ichwati Aulia, 2023^52^ | survey | Indonesia^UMIC^ | NR | N=86 | General Hospital | Healthcare Professionals | Problem-focused, emotion-focused, and avoidance coping | Social Support, Communication, and self-management | Religiosity-based adaptation | The study found significant effects of coping strategies on resilience (p=0.008). Religious coping through prayer and belief in God was widely used, along with social support and adaptability. |
| Mealer, 2013^53^ | Cross-Sectional | South Korea^UMIC^ | 22 - 55 | N=240 (M=20, F=220) | General Hospital | Nurses | Problem-focused coping mechanisms | Social Support, Communication, and self-management | Optimism | Highly resilient ICU nurses (22%) showed significantly lower rates of PTSD, burnout, anxiety, and depression. They demonstrated better adaptation to work stress through optimism, cognitive flexibility, and supportive social networks. |
| Lee, 2015^54^ | Cross-Sectional | South Korea^UMIC^ | 22 - 55 | N=174 (M=14, F=160) | Not defined | Nurses | Positive coping | Social Support, Communication, and self-management | Problem-solving | ED nurses used more positive coping strategies (mean=2.19) than negative ones (mean=1.20). Main positive strategies included cognitive reframing, seeking support, and problem-solving. Experience level influenced coping effectiveness. |
| **Lower-Middle Income Countries** | | | | | | | | | | |
| Yasmin, 2022^55^ | Quasi Experimental Study | Pakistan^LMIC^ | 20 - 35 | N=50 (F=50) | Territory | Nurses | CBT (Cognitive Behavioral Therapy) | Cognitive-behavioral intervention and Mindfulness-based approaches | Educational-interventional | After CBT intervention, burnout scores significantly decreased (32.14 to 23.96, p<0.001) and resilience scores increased (25.40 to 38.16, p<0.001) among critical care nurses. Seventy percent showed improved burnout scores and 82% demonstrated strong resilience levels post-intervention. |
| Ashfaq, 2023^56^ | Cross-Sectional | Pakistan^LMIC^ | 22 - 55 | N=345 (M=132, F=213) | Secondary | Allied health professionals | Organizational Support | Organization and Institutional Approaches | Resource Conservation | Perceived organizational support influenced work engagement through well-being mediation (β=0.06, SE=0.02, CI:0.021-0.107). Resilience moderated this relationship - stronger effect under high resilience (+1SD) compared to low resilience (-1SD). Employee well-being mediated organizational support and work engagement. |
| Akhtar, 2024^57^ | Quasi Experimental Study | Pakistan^LMIC^ | 20 - 50 | N=60 | Territory | Nurses | Problem-solving and Stress management programs | Social Support, Communication, and self-management | Assessment | Problem-solving techniques significantly reduced nurses' stress levels from 2.13 (pre-test) to 1.58 (post-test) in experimental group. Stress management program was effective for emergency nurses. |
| Joker, 2022^58^ | Cross-Sectional | Iran^LMIC^ | 33.7 (2.72) years | N=6914 (M=1499, F=5415) | Territory | Nurses | Resilience | Social Support, Communication, and self-management | Adaptability | Nurses in COVID-19 units had lower resilience (82.5) and professional commitment (45) compared to general unit nurses (93.5 and 55.5 respectively). Strong positive correlation found between resilience and professional commitment (r=0.74, p<0.001 for COVID-19 units). |
| Mirzaei Dahka, 2022^59^ | Cross-Sectional | Iran^LMIC^ | 35.80 (8.83) years | N=432 (M=53, F=379) | Territory | Nurses | Resilience | Social Support, Communication, and self-management | Adaptation | 57.2% nurses showed psychological distress. Higher resilience significantly reduced mental health problems (β=-0.49). |
| Jadidi, 2024^60^ | Qualitative | Iran^LMIC^ | 36.54 (3.51) years | N=14 (M=8, F=6) | Not defined | Healthcare Professionals | Group therapy | Organization and Institutional Approaches | Communication | Group therapy intervention significantly reduced job stress scores from 178.76 to 135.62 across all components (workload, role inadequacy, role duality, role domain, responsibility, physical environment). |
| Fatemi, 2024^61^ | RCT | Iran^LMIC^ | 37.68 (7.08) years | N=77 (M=24, F=53) | Territory | Nurses | Mindfulness-Based Stress Reduction | Cognitive-behavioral intervention and Mindfulness-based approaches | NR | MBSR intervention significantly improved workplace well-being (especially intrapersonal dimension) and empathy levels (particularly perspective-taking) among psychiatric nurses in Iran. |
| Talebiazar, 2024^62^ | RCT | Iran^LMIC^ | 30.73 years | N=60 (M=29, F=31) | Secondary | Nurses | Mindfulness-Based Stress Reduction training | Cognitive-behavioral intervention and Mindfulness-based approaches | NR | MBSR intervention significantly reduced occupational stress and burnout among geriatric nurses compared to control group, including lower scores in death/dying stress, conflicts with staff, workload stress, and treatment uncertainty (p<0.001). |
| Chahbounia, 2023^63^ | Quantitative | Morocco^LMIC^ | NR | N=7 | Secondary | Nurses | Transtheoretical Coaching Model | Cognitive-behavioral intervention and Mindfulness-based approaches | Resource Anchoring | After coaching intervention, nurses' mean stress management knowledge/skills scores improved from 3.71 to 6.57 (p=0.016). The transtheoretical coaching model combining different approaches (cognitive behavioral, humanistic, etc.) helped nurses develop stress management strategies through building confidence and skills. |
| Imamura, 2021^64^ | RCT | Vietnam^LMIC^ | 21 - 55 | N=951 (M=143, F=808) | Territory | Nurses | Smartphone-based internet cognitive behavioral therapy modules including behavioral activation, cognitive restructuring, problem-solving as stress management strategy | Technology-based intervention | Self-guided (The program was fully automated and self-guided, allowing participants to study and practice CBT skills independently) | Fixed-sequence iCBT program showed significant improvement in depression symptoms at 3-months (d=-0.18). Program achieved high completion rates (>83%). Satisfaction and usefulness ratings were high for both program types. |
| Boateng, 2021^65^ | Cross-Sectional | Ghana^LMIC^ | 29 - 58 years | N=40 (M=13, F=27) | Territory | Nurses | Problem-focused (42.5% of respondents used problem-focused coping strategy as their primary mechanism to relieve burnout) | Social Support, Communication, and self-management | Emotional support (22.5% of respondents relied on emotional support from family/friends as a resilience behavior) | Problem-focused coping was most common (42.5%), followed by emotional support (22.5%), humour (15%), and music (12.5%). Adequate staff motivation (30%) and counseling units (27.5%) were recommended institutional coping strategies. |
| Valipour, 2020^66^ | Quasi Experimental Study | Iran^LMIC^ | 20 - 25 | N=80 | Secondary | Nurses | Cognitive-behavioral training | Cognitive-behavioral intervention and Mindfulness-based approaches | Relaxation techniques | A four-session stress management training intervention significantly reduced stress levels in novice nurses (from 3.54 to 3.10) by teaching relaxation, cognitive restructuring, and coping strategies vs control group. |
| Hasan, 2018^67^ | Cross-Sectional | Egypt^LMIC^ | 20 - 40 | N=70 (M=26, F=44) | Territory | Nurses | Self-assurance and self-confidence coping mechanisms | Social Support, Communication, and self-management | Problem-focused coping approach | Psychiatric nurses showed moderate-to-high stress levels (62.8%). Self-assurance coping strategies were most used. Depression was prevalent (75.7%). Work stress significantly correlated with depression (r=0.71, p=.01). |
| Alosaimi, 2016^68^ | Cross-Sectional | Saudi Arabia^HIC^ | 27.9 (4) years | N=147 (M=82, F=65) | Territory | Administrative employees (non-clinical) | Adaptive strategies | Social Support, Communication, and self-management | Stress-adjustment strategies | Residents showed lower adaptive coping strategies (p=0.044), and similar maladaptive coping compared to administrative staff. Stress levels correlated positively with maladaptive coping (rho=0.535) but not with adaptive strategies (rho=0.183). |
| Didehvar, 2016^69^ | Quasi Experimental Study | Iran^LMIC^ | 31.4 (5) years | N=62 | Not defined | Midwives | PRECEDE-PROCEED training model | Cognitive-behavioral intervention and Mindfulness-based approaches | Problem-oriented and emotion-oriented methods | PRECEDE-PROCEED based stress management training significantly decreased stress levels and increased coping behaviors in both nurses and midwives. The training included problem-solving techniques, assertiveness skills, relaxation, and stressor documentation. |
| Pahlavanzadeh, 2016^70^ | RCT | Iran^LMIC^ | 31.71 years | N=65 (F=65) | Territory | Nurses | stress management program including physical methods, muscular relaxation, cognitive restructuring, critical thinking skills and communication training | Cognitive-behavioral intervention and Mindfulness-based approaches | Multi-component adaptation through physical and psychosocial coping skills development | The stress management program significantly improved nursing care quality scores across physical, psychosocial, and communicational dimensions immediately after and 1-month post-intervention (P<0.001) compared to control group. |
| Sailaxmi, 2015^71^ | Quasi Experimental Study | India^LMIC^ | 24 - 50 | N=53 (M=5, F=48) | Not defined | Nurses | Problem-solving | Social Support, Communication, and self-management | Communication skills | The 10-session stress management program significantly reduced nurses' stress levels through problem-solving, time management, communication skills, assertiveness training, responding to criticism, and negotiation skills training. |
| Pahlevani, 2015^72^ | Quasi Experimental Study | Iran^LMIC^ | 24 - 45 | N=40 (M=3, F=37) | Secondary | Nurses | Cognitive-behavioral stress management training | Cognitive-behavioral intervention and Mindfulness-based approaches | Problem-solving | Ten sessions of cognitive-behavioral stress management training significantly improved nurses' psychological well-being. The program included problem-solving, time management, communication skills, assertiveness training, and stress management strategies (p<0.001). |
| PAN, 2015^73^ | Cross-Sectional | India^LMIC^ | 20 - 50 | N=300 (M=20, F=280) | Secondary | Nurses | Problem-focus | Social Support, Communication, and self-management | Career planning | Family factor was most important for stress-coping (weight=0.356), followed by personal attributes (0.275). Top determinants: children's education (0.13), career planning (0.10), family health (0.09), benefits (0.08). |
| Yeboaa, 2024^74^ | cross sectional | Ghana^LMIC^ | 32 | N=394 (M=193, F=201) | General Hospital | Frontline healthcare workers | Problem-focused | Social Support, Communication, and self-management | Self-efficacy | Frontline health workers with high coping self-efficacy showed reduced stress levels. Individual efficacy in stopping negative emotions and thoughts served as a protective strategy against stress during COVID-19. |
| **Low Income Country** | | | | | | | | | | |
| Kleinau, 2024^75^ | RCT | Malawi^LIC^ | <30 years | N=1584 | Primary | Healthcare Professionals | Virtual chatbot which include CBT and Positive Psychology which include psychoeducation, cognitive restructuring, behavioral activation, gratitude, and practical exercises (breathing, relaxation, and meditation). | Technology-based intervention | Self-assessment | Virtual chatbot (Vitalk) significantly reduced depression (-0.68), anxiety (-0.44), burnout (-0.58) and increased resilience (1.47) compared to control group. |

**References**

1. Brooks, E., Wilkinson, J. M. & Popkess-Vawter, S. Promoting situational support for nurses in practice. *Image J. Nurs. Sch.* **26**, 305–307 (1994).

2. Begley, T. M. Coping strategies as predictors of employee distress and turnover after an organizational consolidation: A longitudinal analysis. *J. Occup. Organ. Psychol.* **71**, 305–329 (1998).

3. Mackenzie, C. S., Poulin, P. A. & Seidman-Carlson, R. A brief mindfulness-based stress reduction intervention for nurses and nurse aides. *Appl. Nurs. Res.* **19**, 105–109 (2006).

4. Wallace, E. V. Helping nurses manage their stress by integrating health promotion. *Californian J. Health Promot.* **4**, 9–12 (2006).

5. Hersch, R. K. *et al.* Reducing nurses’ stress: A randomized controlled trial of a web-based stress management program for nurses. *Appl. Nurs. Res.* **32**, 18–25 (2016).

6. Jordan, T. R., Khubchandani, J. & Wiblishauser, M. The impact of perceived stress and coping adequacy on the health of nurses: A pilot investigation. *Nurs. Res. Pract.* **2016**, 5843256 (2016).

7. Magtibay, D. L., Chesak, S. S., Coughlin, K. & Sood, A. Decreasing stress and burnout in nurses: Efficacy of blended learning with Stress Management and resilience training program. *J. Nurs. Adm.* **47**, 391–395 (2017).

8. Mealer, M. *et al.* Designing a resilience program for critical Care Nurses. *AACN Adv. Crit. Care* **28**, 359–365 (2017).

9. Blackburn, L. M., Thompson, K., Frankenfield, R., Harding, A. & Lindsey, A. The THRIVE© program: Building oncology nurse resilience through self-care strategies. *Oncol. Nurs. Forum* **47**, E25–E34 (2020).

10. Dutton, S. & Kozachik, S. L. Evaluating the outcomes of a web-based stress management program for nurses and nursing assistants. *Worldviews Evid. Based. Nurs.* **17**, 32–38 (2020).

11. Hilcove, K. *et al.* Holistic nursing in practice: Mindfulness-based yoga as an intervention to manage stress and burnout. *J. Holist. Nurs.* **39**, 29–42 (2021).

12. Shechter, A. *et al.* Psychological distress, coping behaviors, and preferences for support among New York healthcare workers during the COVID-19 pandemic. *Gen. Hosp. Psychiatry* **66**, 1–8 (2020).

13. Croghan, I. T. *et al.* Stress, resilience, and coping of healthcare workers during the COVID-19 pandemic. *J. Prim. Care Community Health* **12**, 21501327211008450 (2021).

14. Jochen, K. C. *et al.* Randomized controlled trial of the “WISER” intervention to reduce healthcare worker burnout. *Journal of Perinatology* **41**, 2225–2234 (2021).

15. McLean, C. P. *et al.* Helping the helpers: Adaptation and evaluation of stress First Aid for healthcare workers in the Veterans Health Administration during the COVID-19 pandemic. *Workplace Health Saf.* **71**, 162–171 (2023).

16. Torres, K. A. *et al.* Creative arts intervention to reduce burnout and decrease psychological distress in healthcare professionals: A qualitative analysis. *Arts Psychother.* **83**, 102021 (2023).

17. Sos, T. & Melton, B. A qualitative analysis of a mindfulness-based stress management program to reduce stress and burnout for health care staff. *Holist. Nurs. Pract.* **39**, 25–31 (2025).

18. Lin, L., He, G., Yan, J., Gu, C. & Xie, J. The effects of a modified mindfulness-based stress reduction program for nurses: A randomized controlled trial. *Workplace Health Saf.* **67**, 111–122 (2019).

19. Huang, F. *et al.* Resilience of frontline nurses during the COVID pandemic in China: A qualitative study. *Nurs. Health Sci.* **23**, 639–645 (2021).

20. Li, W.-Q. *et al.* Resilience, coping style, and COVID-19 stress: effects on the quality of life in frontline health care workers. *Psychol. Health Med.* **27**, 312–324 (2022).

21. Jiang, J. *et al.* Psychological resilience of emergency nurses during COVID-19 epidemic in Shanghai: A qualitative study. *Front. Public Health* **10**, 1001615 (2022).

22. Chen, X., Zhang, Y., Zheng, R., Hong, W. & Zhang, J. Latent profiles of nurses’ moral resilience and compassion fatigue. *Nurs. Ethics* **31**, 635–651 (2024).

23. Sun, W. *et al.* Perceived stress and coping strategies for frontline nurses caring for COVID-19 patients: A path analysis. *West. J. Nurs. Res.* **47**, 15–23 (2025).

24. Zhang, X. *et al.* Mediating role of resilience on burnout to well-being for hospital nursing staff in Northeast China: a cross-sectional study. *BMJ Open* **14**, e081718 (2024).

25. Rinaldi, A., Tecchio, R., Perugino, S. & De Luca, A. The educational intervention “Focusing” as a strategy to stress reduction among health care workers: a pilot study in an Italian teaching hospital. *Ann. Ig.* **31**, 236–243 (2019).

26. Collantoni, E. *et al.* Psychological distress, fear of COVID-19, and resilient coping abilities among healthcare workers in a tertiary first-line hospital during the Coronavirus pandemic. *J. Clin. Med.* **10**, 1465 (2021).

27. Guerra, F. *et al.* Quality of life and stress management in healthcare professionals of a Dental care setting at a teaching hospital in Rome: Results of a randomized controlled clinical trial. *Int. J. Environ. Res. Public Health* **19**, 13788 (2022).

28. Vallone, F., Cattaneo Della Volta, M. F. & Zurlo, M. C. Stress dimensions, patterns of coping, and psychopathological risk among nurses: a person-centred approach. *BMC Nurs.* **23**, 569 (2024).

29. Martínez-Zaragoza, F. *et al.* When and how do hospital nurses cope with daily stressors? A multilevel study. *PLoS One* **15**, e0240725 (2020).

30. Sánchez-Zaballos, M. & Mosteiro-Díaz, M. P. Resilience among professional health workers in emergency services. *J. Emerg. Nurs.* **47**, 925-932.e2 (2021).

31. Buch-Vicente, B. *et al.* Coping strategies used by health-care workers during the SARS-COV2 crisis. A real-world analysis. *Psychiatry Res.* **317**, 114915 (2022).

32. Chen, C.-K., Lin, C., Wang, S.-H. & Hou, T.-H. A study of job stress, stress coping strategies, and job satisfaction for nurses working in middle-level hospital operating rooms. *J. Nurs. Res.* **17**, 199–211 (2009).

33. Lee, W.-L., Tsai, S.-H., Tsai, C.-W. & Lee, C.-Y. A study on work stress, stress coping strategies and health promoting lifestyle among district hospital nurses in Taiwan. *J. Occup. Health* **53**, 377–383 (2011).

34. Watanabe, N. *et al.* Brief mindfulness-based stress management program for a better mental state in working populations - Happy Nurse Project: A randomized controlled trial✰✰. *J. Affect. Disord.* **251**, 186–194 (2019).

35. Isa, K. Q. *et al.* Strategies used to cope with stress by emergency and critical care nurses. *Br. J. Nurs.* **28**, 38–42 (2019).

36. Betke, K., Basińska, M. A. & Andruszkiewicz, A. Sense of coherence and strategies for coping with stress among nurses. *BMC Nurs.* **20**, 107 (2021).

37. Understanding nurses’ strategies to handle (Un)wanted nurse calls: A resilience perspective. *Comput. Inform. Nurs.* **35**, 323–324 (2017).

38. Dumarkaite, A. *et al.* The efficacy of the internet-based stress recovery intervention FOREST for nurses amid the COVID-19 pandemic: A randomized controlled trial. *Int. J. Nurs. Stud.* **138**, 104408 (2023).

39. Foster, K. *et al.* Promoting resilience in mental health nurses: A partially clustered randomised controlled trial. *Int. J. Nurs. Stud.* **159**, 104865 (2024).

40. Ma, H.-Y., Chiang, N.-T., Kao, R.-H. & Lee, C.-Y. Health workers’ mindfulness-based stress reduction and resilience during COVID-19 pandemic. *J. Multidiscip. Healthc.* **17**, 3691–3713 (2024).

41. Uccella, L., Mascherona, I., Semini, S. & Uccella, S. Exploring resilience among hospital workers: a Bayesian approach. *Front. Public Health* **12**, 1403721 (2024).

42. Tyler, P. & Cushway, D. Stress, coping and mental well‐being in hospital nurses. *Stress Med.* **8**, 91–98 (1992).

43. Bruneau, B. M. S. & Ellison, G. T. H. Palliative care stress in a UK community hospital: evaluation of a stress-reduction programme. *Int. J. Palliat. Nurs.* **10**, 296–304 (2004).

44. Rosenthal, S. L., Schmid, K. D. & Black, M. M. Stress and coping in a NICU. *Res. Nurs. Health* **12**, 257–265 (1989).

45. von Baeyer, C. & Krause, L. Effectiveness of stress management training for nurses working in a burn treatment unit. *Int. J. Psychiatry Med.* **13**, 113–126 (1983).

46. Dabou, E. A. R., Ilesanmi, R. E., Mathias, C. A. & Hanson, V. F. Work-related stress management behaviors of nurses during COVID-19 pandemic in the United Arab Emirates. *SAGE Open Nurs.* **8**, 23779608221084972 (2022).

47. Munawar, K. & Choudhry, F. R. Exploring stress coping strategies of frontline emergency health workers dealing Covid-19 in Pakistan: A qualitative inquiry. *Am. J. Infect. Control* **49**, 286–292 (2021).

48. Öksüz, E. *et al.* Resilience in nurses in terms of perceived social support, job satisfaction and certain variables. *J. Nurs. Manag.* **27**, 423–432 (2019).

49. Sis Çelik, A. & Yarali, S. The effect of laughter yoga on the psychological resilience and sleep quality of nurses during the pandemic: A randomized controlled trial. *Altern. Ther. Health Med.* **29**, 146–152 (2023).

50. Umann, J., da Silva, R. M., Benavente, S. B. T. & Guido, L. de A. The impact of coping strategies on the intensity of stress on hemato-oncology nurses. *Rev. Gaucha Enferm.* **35**, 103–110 (2014).

51. Celestino, L. C., Leal, L. A., Lopes, O. C. A. & Henriques, S. H. Work-related psychosocial risks of the Family Health nurse and management strategies. *Rev. Esc. Enferm. USP* **54**, e03602 (2020).

52. Ichwati Aulia, C., Mudatsir, M. & Mawarpury, M. The role of coping strategies in the resilience of health workers during covid-19 pandemic. *E3S Web Conf.* **447**, 05003 (2023).

53. Mealer, M. *et al.* The presence of resilience is associated with a healthier psychological profile in intensive care unit (ICU) nurses: results of a national survey. *Int. J. Nurs. Stud.* **49**, 292–299 (2012).

54. Lu, D.-M. *et al.* Occupational stress and coping strategies among emergency department nurses of China. *Arch. Psychiatr. Nurs.* **29**, 208–212 (2015).

55. Yasmin, K., Yaqoob, A. & Sarwer, H. Effect of cognitive behavioral therapy (CBT) based intervention on resilience and burnout among staff nurses working in critical care departments. *Pakistan Journal of Medical and Health Sciences* **16**, 295–298 (2022).

56. Ashfaq, F., Abid, G., Ilyas, S. & Binte Mansoor, K. Perceived organisational support and work engagement among health sector workers during the COVID-19 pandemic: a multicentre, time-lagged, cross-sectional study among clinical hospital staff in Pakistan. *BMJ Open* **13**, e065678 (2023).

57. Akhtar, N. Stress management among emergency nurses: Knowing the problem-solving mechanism in mayo hospital, Lahore, Pakistan. *Journal of Population Therapeutics & Clinical Pharmacology* 1730–1734 (2024) doi:10.53555/jptcp.v31i6.6754.

58. Joker, Z., Torabizadeh, C., Movahednia, Z., Hashemizadeh Fard Haghighi, L. & Bijani, M. Resilience and professional commitment in nurses in practice in COVID-19 units compared to other nurses. *Neuropsychiatr. Neuropsychol.* **17**, 108–114 (2022).

59. Mirzaei Dahka, S. *et al.* Mental health and resilience among nurses in the COVID-19 pandemic: A web-based cross-sectional study. *Iran. J. Psychiatry* **17**, 35–43 (2022).

60. Jadidi, A., Irannejad, B., Safarabadi, M. & Zand, S. Evaluation of stress management effectiveness using the action research approach on the job stress of pre-hospital emergency staff. *Int. J. Afr. Nurs. Sci.* **20**, 100702 (2024).

61. Fatemi, J., Vagharseyyedin, S. A. & Askari-Noghani, A. The impact of mindfulness-based stress reduction on workplace well-being and empathy levels among nurses working in psychiatric wards in Iran: A controlled trial. *Issues Ment. Health Nurs.* **45**, 1082–1089 (2024).

62. Talebiazar, N. *et al.* The impact of mindfulness-based stress reduction training on the occupational stress and burnout experienced by nurses in geriatric wards? A randomized controlled trial. *Geriatr. Nurs.* **58**, 373–381 (2024).

63. Chahbounia, R. & Gantare, A. A pilot study to assess the effect of coaching on emergency nurses’ stress management. *Nurs. Rep.* **13**, 179–193 (2023).

64. Imamura, K. *et al.* Effect of smartphone-based stress management programs on depression and anxiety of hospital nurses in Vietnam: a three-arm randomized controlled trial. *Sci. Rep.* **11**, 11353 (2021).

65. Boateng, Y. A., Osei, S. A., Aboh, I. K. & Druye, A. A. Causes of burnout syndrome and coping strategies among high dependency unit nurses of an institution in the greater Accra region of Ghana. *Nurs. Open* **8**, 3334–3339 (2021).

66. Valipour, S., Aazami, S. & Mozafari, M. The effect of training intervention on the level of stress management skills in novice nurses working at educational hospitals in Ilam Province. *Journal of Cardiovascular Disease Research* **11**, 98–104 (2020).

67. Hasan, A. A., Elsayed, S. & Tumah, H. Occupational stress, coping strategies, and psychological-related outcomes of nurses working in psychiatric hospitals. *Perspect. Psychiatr. Care* **54**, 514–522 (2018).

68. Alosaimi, F. D., Alghamdi, A. H., Aladwani, B. S., Kazim, S. N. & Almufleh, A. S. Work-related stress and stress-coping strategies in residents and administrative employees working in a tertiary care hospital in KSA. *J. Taibah Univ. Med. Sci.* **11**, 32–40 (2016).

69. Didehvar, M. *et al.* The effect of stress management training through PRECEDE-PROCEED model on occupational stress among nurses and midwives at Iran hospital, iranshahr. *J. Clin. Diagn. Res.* **10**, LC01–LC05 (2016).

70. Pahlavanzadeh, S., Asgari, Z. & Alimohammadi, N. Effects of stress management program on the quality of nursing care and intensive care unit nurses. *Iran. J. Nurs. Midwifery Res.* **21**, 213–218 (2016).

71. Sailaxmi, G. & Lalitha, K. Impact of a stress management program on stress perception of nurses working with psychiatric patients. *Asian J. Psychiatr.* **14**, 42–45 (2015).

72. Pahlevani, M. *et al.* Effectiveness of stress management training on the psychological well-being of the nurses. *J. Med. Life* **8**, 313–318 (2015).

73. F C Pan, F. Using analytic hierarchy process to identify the nurses with high stress-coping capability: model and application. *Iran. J. Public Health* **43**, 273–281 (2014).

74. Yeboaa, J. B., Agbemafle, I. & Kwame, A. C. Perceived Stress and Coping Mechanisms Among Frontline Health Workers During the Covid-19 Pandemic in the Greater-Accra Region. in *A Cross-Sectional Study* (Ghana, 2024).

75. Kleinau, E. *et al.* Effectiveness of a chatbot in improving the mental wellbeing of health workers in Malawi during the COVID-19 pandemic: A randomized, controlled trial. *PLoS One* **19**, e0303370 (2024).
